# Take the shortcut – direct conversion of somatic cells into induced neural stem cells and their biomedical applications

**DOI:** 10.1002/1873-3468.13656

**Published:** 2019-12-01

**Authors:** Anita Erharter, Sandra Rizzi, Jerome Mertens, Frank Edenhofer

**Affiliations:** ^1^ Department of Molecular Biology & CMBI Genomics, Stem Cell Biology & Regenerative Medicine Leopold‐Franzens‐University Innsbruck Austria; ^2^ Institute of Pharmacology Medical University Innsbruck Austria

**Keywords:** cell therapy, cellular reprogramming, direct conversion, disease modeling, induced neural stem cells, induced pluripotent stem cells, neuronal cells, regionalization, transdifferentiation, transplantation

## Abstract

Second‐generation reprogramming of somatic cells directly into the cell type of interest avoids induction of pluripotency and subsequent cumbersome differentiation procedures. Several recent studies have reported direct conversion of human somatic cells into stably proliferating induced neural stem cells (iNSCs). Importantly, iNSCs are easier, faster, and more cost‐efficient to generate than induced pluripotent stem cells (iPSCs), and also have a higher level of clinical safety. Stably, self‐renewing iNSCs can be derived from different cellular sources, such as skin fibroblasts and peripheral blood mononuclear cells, and readily differentiate into neuronal and glial lineages that are indistinguishable from their iPSC‐derived counterparts or from NSCs isolated from primary tissues. This review focuses on the derivation and characterization of iNSCs and their biomedical applications. We first outline different approaches to generate iNSCs and then discuss the underlying molecular mechanisms. Finally, we summarize the preclinical validation of iNSCs to highlight that these cells are promising targets for disease modeling, autologous cell therapy, and precision medicine.

## Graphical abstract

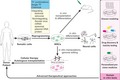

## Abbreviations

**CIP**, congenital insensitivity to pain

**CIPN**, chemotherapy‐induced peripheral neuropathy

**CNS**, central nervous system

**CNVs**, copy number variations

**EGF**, epidermal growth factor

**FGF2**, fibroblast growth factor

**iNSCs**, induced neural stem cells

**iPSCs**, induced pluripotent stem cells

**LIF**, leukemia inhibitory factor

**MEFs**, mouse embryonic fibroblasts

**MET**, mesenchymal‐to‐epithelial transition

**NSCs**, neural stem cells

**PNS**, peripheral nervous system

**SeV**, Sendai virus

**TFs**, transcription factors

**VPA**, valproic acid

## Take the shortcut: from iPSC to iNSC

Forced expression of lineage‐specific transcription factors (TFs) has been shown to reprogram the developmental potential of various somatic cell types e.g. by inducing pluripotency 1. The seminal breakthrough of fibroblast reprogramming into iPSCs offers a novel experimental tool to generate patient‐specific cells for developmental studies and diverse biomedical applications, such as disease modeling and cell therapy. Since then, efficiency and robustness of reprogramming protocols have been substantially improved, but the derivation of patient‐specific iPSCs remains a lengthy and cumbersome procedure. Moreover, clinical applications require subsequent redifferentiation of iPSCs into the cell type of interest, which is inefficient and risky because transplanted undifferentiated iPSCs are tumorigenic. In the shadow of iPSC‐type reprogramming, second‐generation reprogramming paradigms have been developed to bypass the pluripotency state and obtain multipotent precursors/stem cells by direct conversion of somatic cells. In this way, TF‐driven reprogramming of mouse fibroblasts directly yields other somatic cell types, such as induced neurons 2, 3, 4, 5, 6, cardiomyocytes 7, 8, hepatocyte‐like cells 9, blood 10, neural progenitors 11, 12, and neural stem cells (NSCs) 13, 14. This review focuses on the approaches to directly convert mouse and human somatic cells into NSCs, designated as induced NSCs (iNSCs). Similar to both NSCs purified from primary tissue and iPSC‐derived NSCs, iNSCs are defined by their potential to give rise to neurons and glial cells, their self‐renewal, clonal growth, marker expression profile, and epigenetic status. In particular, iNSCs differ from induced neurons (iN; covered by Traxler *et al*., 15 same issue) and from iPSCs for their proliferative capacities and their neural‐lineage‐restricted differentiation potential, respectively.

**Figure 1:**
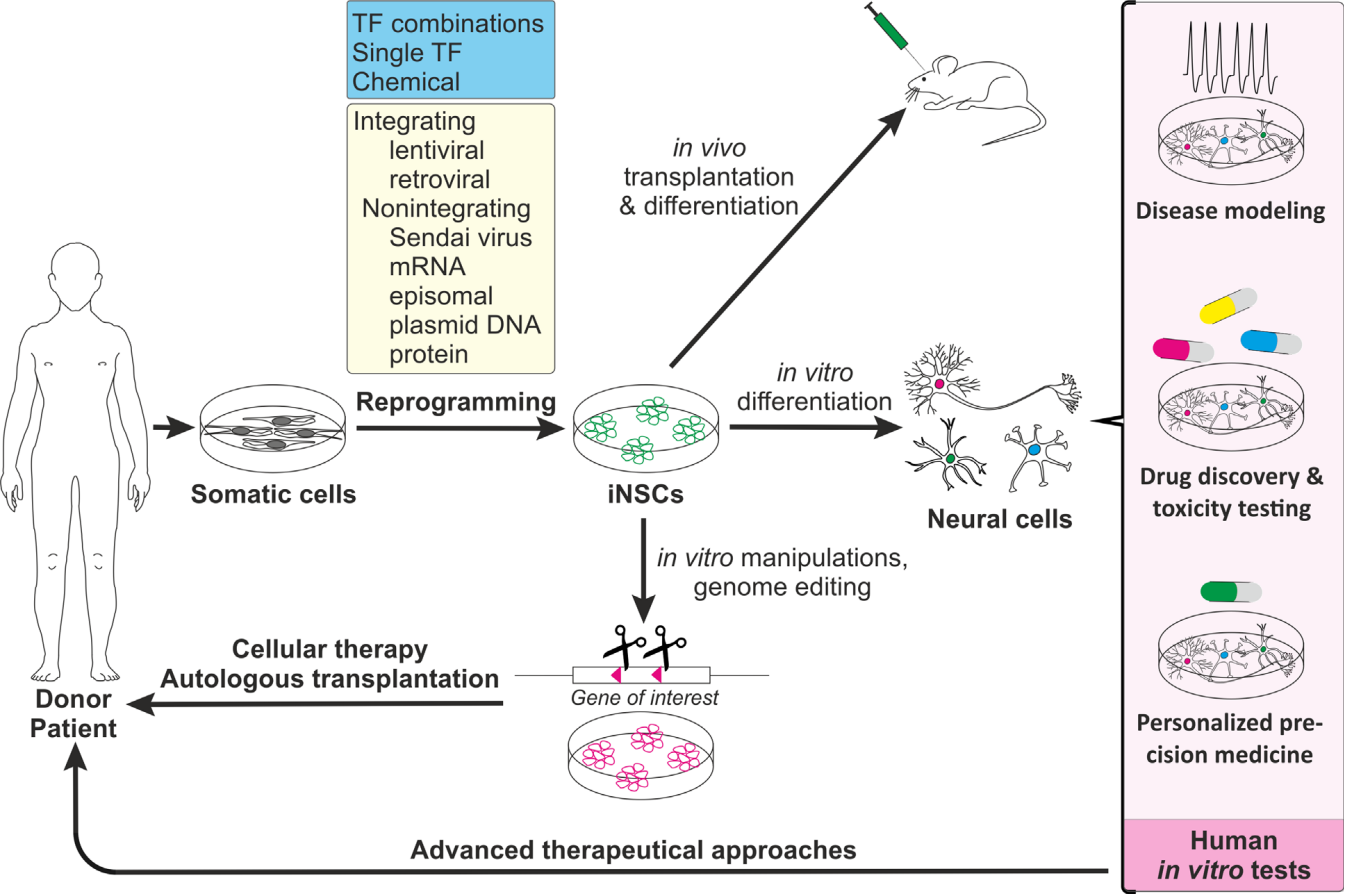
Induced neural stem cells generation and biomedical applications.

Induced neural stem cells can be generated by direct conversion of somatic cells in various ways, using a single TF, a set of TFs, and/or a cocktail of pharmacological compounds, either alone or in combination (Table 1, Fig. 1). The DNA encoding TFs can be integrated into the target cells’ genome or be expressed as nonintegrating episomes. Notably, the applied protocol determines the reprogramming efficiency and the properties of the resulting iNSCs. In this review, we outline the different combinations of TFs in use and the impact of media supplements that enhance reprogramming. Moreover, we discuss recent studies giving insight into the molecular mechanisms leading to cell cycle stabilization and stepwise acquisition of NSC markers with the concomitant loss of somatic cell markers.

**Table 1:** Overview of direct conversion techniques for the derivation of iNSCs. LeV, lentivirus; LeV‐i, inducible LeV; LeV‐i/e, inducible/excisable LeV; ReV, retrovirus; TTFs, tail tip fibroblasts; AA, ascorbic acid; Alk5, Alk5‐inhibitor II; CHIR, CHIR99021; RA, retinoic acid; Hh, hedgehog agonist 1.5; Pur, purmorphamine; TC, tranylcypromine; TZ, thiazovivin; SB, SB431542; LDN, LDN193189; –, not reported or not applicable; ●, applicable

Vehicle	Transcription factor(s)					Cellular source	Conversion efficiency	Conversion supplements/pharmacological compounds							Self‐renewal (Passages)	Reference
	OCT4	SOX2	KLF4	cMYC	Others			EGF	FGF2	LIF	CHIR	SB	LDN	Others		
ReV		●	●	●	Brn2, ±E47/Tcf3	Mouse fibroblasts	1–3(4F)/2–5(5F) colonies of 5 × 10^4^ cells	●	●						> 130	14
ReV + TAT‐protein		●	●	●	Oct4‐protein	Mouse embryonic fibroblasts	7–11 colonies of 1.3 × 10^5^ cells			●					> 50	13
LeV	●					Human cord blood	12 colonies of 5 × 10^4^ cells				●	●	●	Noggin	> 10	30
LeV‐i	●	●	●	●		Mouse embryonic fibroblasts, TTFs	0.07%		●	●				FGF4	3–5	11
LeV‐i					ZFP521	Human fetal/neonatal/adult fibroblasts	0.4–0.7%				●	●		VPA	> 60	36
LeV‐i					Ptf1α	Mouse embryonic fibroblasts, human foreskin fibroblasts	0.5%	● ●							> 32	38
LeV‐i/e		●	●		BRN2, ZIC3	Human peripheral blood, fetal/adult fibroblasts	0.015–0.166%				●			Alk5, pur, TC	> 40	28
Plasmid		●			PAX6	Human dermal fibroblasts	0.05%	●	●					VPA	–	35
Episomal	●	●	●	●	NANOG, LIN28, SV40LT	Human peripheral blood	–			●	●	●			> 60	73
Episomal		●	●	●	Brn2	Mouse embryonic fibroblasts	–	●	●						> 40	58
Episomal + miRNA	●	●	●		SV40LT + miR‐302–367	Human urine epithelial	0.2%				●			TZ, DMH1	> 11	57
SeV	●	●	●	●		Human postnatal/adult fibroblasts	0.03–0.08%			●	●	●			> 20	25
SeV		●		●		Human peripheral blood	0.08–0.66%			●	●			Pur, A83‐01, AA	> 20	48
TAT‐Protein		●				Human foreskin fibroblasts	2 colonies of 5 × 10^6^ cells			●	●	●		Pur, VPA	> 20	33
mRNA		●				Human cord blood, adult fibroblasts	0.015%	–							> 50	34
Chemical						Mouse embryonic fibroblasts, TTFs, human urine epithelial	–	CHIR, VPA, RepSox							At least 13	59
Chemical						Mouse embryonic fibroblasts, TTFs	–	FGF2, CHIR, LDN‐19319, A83‐01, RA, Hh, RG108, Parnate, SMER28							Up to 20	60
Chemical						Mouse embryonic fibroblasts, TTFs	–	CHIR, VPA, RepSox, Il‐6, FGF5, LIF							At least 24	62

In regard to potential therapeutic and biomedical applications of second‐generation reprogramming protocols, transgene‐free iNSCs that do not harbor permanent and potentially functionally harmful genetic modifications would be highly desirable. Hence, we review the cutting‐edge nonintegrating methods for the delivery of TFs and comprehensively discuss the distinct outcomes of different iNSC conversion protocols in terms of cellular markers, differentiation potential, and physiological functions of the neural progeny. Moreover, epigenetic marks that are present in the somatic cell population, including those correlating with aging, are differently erased by the reprogramming pathways. Instead, iPSCs display an almost complete epigenetic reset, whereas iNs maintain most of the epigenetic marks, a phenomenon that is comprehensively addressed by Denoth‐Lippuner and Jessberger in this issue 16. iNSCs can be produced faster and more cost‐efficient than iPSC‐derived NSCs, and also harbor less tumor formation potential. Thus, in this review, we also discuss promising biomedical applications of iNSCs as models for diseases like Alzheimer's and Huntington's disease, as well as spinocerebellar ataxia type III. Finally, we summarize the current status of preclinical studies that exploit iNSCs for the treatment of spinal cord injury, acute stroke, Parkinson's disease, and multiple sclerosis.

## Transcription factors employed in iNSC generation

Direct conversion of somatic cells into iNSCs was achieved by employing TFs in combination with media supplements, such as small molecules and growth factors needed to stabilize generated iNSCs. In contrast to iPSC generation, the selection of TFs that enable the conversion into iNSCs is quite diverse (Table 1). The majority of the studies involve at least one of the Yamanaka reprogramming factors [Oct4, Sox2, Klf4, and c‐Myc (OSKM) factors], which emphasizes their potency as pioneering factors enabling other neurogenic TFs to access the chromatin 17, 18, 19, 20, 21, 22. In 2011, an initial study published by Kim *et al*. 11 reported the direct conversion of mouse embryonic fibroblasts (MEFs) into a transiently proliferating population of neural stem progenitors. To this end, MEFs were retrovirally transduced with the Yamanaka factors in the presence of three growth factors (EGF, FGF2, and FGF4), which favor trapping and expansion of neural progenitors. However, those converted neural progenitors divided only a few times, indicating that the OSKM factors together and neurogenic media are insufficient to generate stable iNSCs. In 2012, Thier *et al*. 13 obtained stably proliferating mouse iNSCs by time‐restricted expression of the OSKM factors. The authors discovered that the continuous expression of Oct4 favors the generation of iPSCs at the expenses of iNSC. Consequently, they decided to induce Oct4 only at the beginning of the conversion process while maintaining SKM under the control of constitutively active promoters. Time‐restricted expression of Oct4 during the first five days of reprogramming was achieved by either doxycycline‐induced expression, mRNA transfection, or protein transduction 13. Interestingly, Han *et al*. 14 published at the same time the generation of iNSCs using constitutively active SKM together with Brn2 (BSKM). Like Oct4, Brn2 belongs to the family of POU TFs harboring a well‐conserved bipartite DNA‐binding domain. So far, about 20 different POU family members have been described in mammals with roles in (early) embryonic development and cell maintenance 23. Furthermore, POU factors bind to chromatin remodeling factors, presumably explaining a pioneering activity 24. The findings by Thier *et al*. 13 and Han *et al*. 14 that a short window of POU factor Oct4 expression or its substitution by POU factor Brn2 yield stably proliferating iNSCs jointly indicate that Oct4 is a key determinant for commitment toward both iPSCs and multipotent iNSCs. Other studies used Sendai‐viral transduction to express OSKM factors and supplementation of leukemia inhibitory factor (LIF), SB431542, and CHIR99021 to generate human iNSCs from human fibroblasts 25, 26. Here, the authors destabilized the Sendai virus (SeV) to control Oct4 expression over time.

Next to the pluripotency‐associated OSKM factors, neurogenic TFs have been also widely exploited. In 2012, Kumar *et al*. 27 generated human fibroblast‐derived iNSCs through retroviral overexpression of Brn2 with Klf4, Sox2, and Zic3 in a medium supplemented with growth factor FGF2. Very recently, Thier *et al*. 28 used the same combination of TFs along with CHIR99021, Alk5 inhibitor II, purmorphamine, and tranylcypromine, to directly convert human fibroblasts, as well as human peripheral blood cells, into early neuroepithelial stem cells. Two studies reported that lentivirus‐mediated expression of OCT4 is sufficient to obtain iNSCs from adult human fibroblasts, neonatal cord, and peripheral blood cells 29, 30. Noteworthy, both studies took advantage of either supplementation with FGF2 or dual SMAD inhibition during the reprogramming process. Dual SMAD inhibition is known to efficiently and rapidly convert pluripotent cells into neural cells 31, potentially indicating that reprogrammed cells might have passed a transient pluripotent stage. Besides Oct4, also Sox2 has been reported to be sufficient for direct conversion of somatic cells into iNSCs 32, 33, 34. As a matter of fact, Sox2 alone or in combination with other TFs is the most frequently employed factor in the available second‐generation reprogramming protocols (Table 1). This is likely owed to its function as master regulator for specification and maintenance of progenitors of the neural tube and the central nervous system (CNS) 35.

Several groups succeeded in direct OSKM‐ and BSKM‐independent conversion of somatic cells into iNSCs 12, 28, 36, 37, 38. Two studies reported the generation of iNSCs from human fibroblasts through inducible lentiviral transduction of Zfp521, a neurogenic TF, of cells cultured under hypoxic conditions in a reprogramming medium that contained valproic acid, CHIR99021, and SB431542 36, 39. Another approach allowed to directly converting mouse and human fibroblasts, including cells derived from AD patients, into iNSCs with an efficiency higher than 0.5% 38. In this case, the authors used a lentiviral vector to overexpress PTF1a, which is a non‐neurogenic basic helix‐loop‐helix TF showing low expression in non‐neural postmitotic precursor cells during embryo development 38. The unexpected reprogramming activity of this non‐neurogenic TF critically depends on its interaction with RBPJ to activate downstream TFs involved in NSC specification. In fact, it causes activation of at least six families of TF genes, including Sox and POU domain family members, thus making possible SOX2 and OCT4 activation during the Ptf1a‐induced transdifferentiation procedure 38.

In 2017, Hou *et al*. 37 reported two reprogramming protocols exploiting either six or seven TFs for direct OSKM‐ and BSKM‐independent conversion of human fibroblasts into iNSCs. To some extent, the establishment of regional identity of iNSCs appeared to depend on which set of TFs was chosen. Expression of six TFs (CBX2, HES1, ID1, TFAP2A, ZFP42, and ZNF423) yielded forebrain, midbrain, and spinal cord iNSCs. In contrast, the seven TFs (FOXG1, GATA3, NR2F1, PAX6, SALL2, TFAP2A, and ZFP42) gave rise to iNSCs of both hindbrain and the peripheral nervous system (PNS) 37. Generation of region‐specific iNSCs was reported also by Lu *et al*. 25 who obtained iNSCs expressing predominantly hindbrain genes, which maintained their identity even after transplantation into mouse forebrain. The reprogramming protocol used in this study relied on infection with SeV carrying OSKM and a reprogramming medium supplemented with LIF, SB431542, and CHIR99021. However, the iNSCs generated by Thier *et al*. 13 using OSKM do not fully correlate with a distinct regional identity, but were reportedly mostly compatible with a mixed ventral fore‐/mid‐/hindbrain identity. In contrast to the study by Lu *et al*., EGF and FGF2 were added to the medium to favor iNSC generation. In conclusion, the reprogramming protocol has a strong influence on the regional identity of the converted iNSCs. This depends on both the choice of TFs and the compounds added to the medium. To date, a great variety of TFs and compounds are known to be able to induce NSC fate. Future studies shall focus on exploring alternative TFs and culture conditions capable of converting somatic cells directly into regionally defined iNSCs that make it possible to obtain distinct iNSC subpopulations for cell transplantation and replacement studies.

## Molecular mechanisms regulating direct conversion of somatic cells into iNSCs

Induced neural stem cells are generated through direct conversion, a process in which cells acquire the expression of NSC markers in a stepwise fashion, theoretically without passing through a pluripotent state 40. However, most protocols for iNSC generation involve the Yamanaka factors OSKM 11, 13, 25, 26, 41, 42, suggesting that cells undergo a transient pluripotent state during the reprogramming process. Various efforts have been made to dissect the trajectory of somatic cells undergoing conversion into iNSCs. Potential pluripotent intermediates can be detected through the analysis of endogenous gene expression of pluripotency master regulators, such as Oct4 and Nanog, silencing of retroviral transgenes, methylation status of promoter regions, and silenced X chromosome reactivation. Employing appropriate lineage tracing tools, OSKM expression during iNSC generation revealed induction of a temporary pluripotent state indicating coupling of OSKM‐mediated iNSC generation to iPSC reprogramming mechanisms 43, 44. One study used an Oct4‐CreER labeling system to trace the Oct4‐expressing progeny 43. The authors report that iNSCs generated using OKSM originate from cells showing transient expression of endogenous Oct4 and that ablation of the Oct4‐expressing cells prevented subsequent iNSC formation. The same group used also BSKM, reportedly unable to induce iPSCs, to induce cell transdifferentiation 14. In contrast to previous observations, Bar‐nur *et al*. 43 surprisingly showed that BSKM was actually able to generate iPSC‐like colonies and hallmarked by activation of endogenous pluripotency genes *Oct4*,* Nanog*, and *Zfp42*, in both embryonic stem cell and NSC media. Furthermore, lineage tracing revealed that also BSKM‐iNSCs had passed a transient Oct4‐positive state. Another study used two lineage tracing systems, Nanog‐CreER and Oct4‐CreER*,* to follow the transdifferentiation of mouse embryonic fibroblasts (MEFs) toward cardiomyocytes or iNSCs 44. The vast majority of transdifferentiated iNSCs underwent a transient pluripotent state during reprogramming. Additionally, just over 90% of the silenced X chromosome was found to be reactivated, as compared to 100% in iPSCs. Together, these studies suggest that conversion of somatic cells into iNSCs using OSKM‐ or BSKM‐mediated pathways involves transient expression of pluripotency‐associated genes and, depending on the culturing conditions applied, subsequent induction of the neural fate. Despite a number of studies pointing to a transient pluripotent state during iNSC conversion, one cannot rule out that direct conversion to multipotency is possible also by bypassing pluripotency. Several lines of evidence support this notion: Velychko *et al*. 45 demonstrated that acquisition of pluripotency during reprogramming depends on the type of expression vectors. While BSKM generates iNSCs without a transient pluripotent state, if expressed individually, polycistronic BSKM expression can induce pluripotency. The authors showed that polycistronic BSKM expression results in a Brn4‐Klf4 fusion protein owing to incomplete cleavage of the F2A peptide that usually leads to two productions of individual proteins post‐translationally. According to their analyses, fusion to Klf4 re‐enabled Brn4 to induce pluripotency. This can be explained by the fact that both Oct4 and Brn4 are members of the POU family of TFs and share similar protein domains. While Oct4 is a known pluripotency inducer, Brn4 alone fails to induce pluripotency because of the functional inadequacy of its POU‐specific domain. This observation would also explain the findings of Thier *et al*. 13 in one of the initial studies of iNSC conversion. The authors deliberately used a tightly regulated system for Oct4 expression while constitutively inducing activity of SKM. They reasoned that this approach combines the strong neuroinductive capacity resulting from high Sox2, Klf4, and c‐Myc levels, while reducing the induction of pluripotency by limiting the expression of Oct4. Notably, the authors stated that transduction of Oct4 protein or mRNA transfection is preferable to transcriptional control. In conclusion, identification of potential pluripotency intermediates during reprogramming procedures is important to distinguish between iPSC‐coupled mechanisms and true transdifferentiation 40, 43, 44.

Apart from potential pluripotency intermediates, transdifferentiated cells might harbor somatic memory. While full rejuvenation to a pluripotent state resets the epigenetic signatures of the cellular source almost completely 46, 47 (and reviewed in this issue 16), little is known about epigenetic memory in iNSCs. Interestingly, Thier *et al*. 28 reported the expression of fibroblast marker COL3A1 in fibroblast‐derived, but not blood‐derived iNSCs. Besides the expression of COL3A1, the authors did not observe any other features of fibroblasts, suggesting that the residual fibroblast memory was not sufficient to hamper neural stem cell identity and function in this case. A recent study included a comprehensive genome‐wide methylome analysis of reprogrammed iNSCs 48. The authors reported that low passage iNSCs display a marked loss of age‐related DNA methylation signatures, which further erode across extended passaging. Moreover, there appears to be a significant clonal variability in terms of epigenetic resetting that needs to be investigated in more detail in the future.

Although there is still some debate on the occurrence of a transitional pluripotent state, as a matter of fact stably converted iNSCs do not contain iPSC contaminants, making them particularly useful for therapeutic applications.

## Generation of transgene‐free iNSCs

Integrating methods usually rely on viral vector systems, widely used and validated tools for the generation of transgenic animal models and cell lines. Retro‐ and lentiviral vectors integrate into the target genome thereby leading to permanent genetic modifications of the host genome and often affect the transcriptome also by driving persistent transgene expression 49. Hence, although several studies successfully used retro‐ or lentiviral vectors to transdifferentiate somatic cells into iNSCs, a transgene factor‐free protocol would be desired to reduce the risk that permanent genetic modifications interfere with the normal function of the obtained iNSCs 50. Thier *et al*. 13 made use of a reprogramming‐competent recombinant cell‐permeant Oct4‐TAT fusion protein to achieve stably expandable and tripotent iNSCs. The TAT sequence from the HIV transactivator of transcription encodes a cell‐penetrating peptide that promotes translocation of a target protein into cells 51, 52. However, apart from Oct4, the other reprogramming factors needed to be activated by conventional retroviral infection. Recently, an inducible lentivirus‐based vector system for iNSC generation has been further optimized by means of a loxP‐flanked expression cassette 53. This enabled subsequent Cre‐mediated transgene removal after iNSC generation. Using this excisable reprogramming vector system, the authors could derive transgene‐free iNSCs by direct conversion of human adult peripheral blood monocytes, as well as dermal and fetal pancreas fibroblasts. While this system combines the high efficacy of lentiviral transduction with minimal permanent genetic modifications, a series of additional attempts have been made to adapt nonintegrating systems to iNSC generation. Mauksch *et al*. reported the generation of iNSCs by nonviral means employing plasmid transfection and recombinant protein transduction using SOX2 and PAX6 54. Another nonintegrating strategy relies on the usage of episomal vectors, which are able to replicate their genome autonomously as extrachromosomal DNA. They can be based on (a) replication of deficient viruses, such as adenovirus and adeno‐associated virus, (b) isolated plasmid replicons of viruses, such as simian virus 40 (SV40) and Epstein–Barr virus (EBV), (c) substituted viral plasmid replicons, such as oriP/EBNA1 of EBV, or (d) chromosomal elements, such as pEPI‐based vectors and artificial chromosomes 55. Also, microRNAs play important roles in the stabilization of reprogrammed cells 56. Wang *et al*. 57 generated integration‐free and expandable iNSCs from human urine‐derived epithelial cells using oriP/EBNA episomal vectors encoding OCT4, SOX2, KLF4, and SV40LT, in combination with the microRNA cluster MIR302‐367. Similar oriP/EBNA of EBV‐based episomal vectors were used to express OKSM and LIN28 in combination with a small hairpin against p53 in adult human fibroblasts 42 or Brn4, Klf4, Sox2, and c‐Myc in mouse embryonic fibroblasts 58. In 2013, Sendai virus (SeV) was employed as a nonintegrating viral vector for the generation of iNSCs 25. SeV is a nonsegmented negative‐sense single‐stranded RNA virus, which primarily infects mammalian cells and replicates exclusively in the host cytoplasm. In recent years, SeV‐based vectors have become increasingly used for the generation of transgene‐free iPSCs and have been consequently adapted for the generation of iNSCs from human and monkey postnatal and adult fibroblasts 25, 26. Though nonintegrating viral vectors are able to generate iNSCs, they exhibit lower reprogramming efficiencies compared to integrating viral systems. Moreover, SeV production is very laborious and costly. Recent studies propose chemical‐based protocols for direct conversion of somatic cells into iNSCs 59, 60, 61, 62. Direct conversion by chemical reprogramming has been shown to reach an efficiency of up to 30% and comes with the advantage of using pharmacologically active compounds that can be controlled in a time‐ and dose‐dependent manner 63, 64. In 2014, Cheng *et al*. 59 reported the generation of iNSCs from mouse fibroblasts and human urinary cells using a cocktail of three small molecules, namely valproic acid (VPA), CHIR99021, and RepSox under hypoxic conditions. VPA is a histone‐deacetylase (HDAC) inhibitor often used in cell reprogramming and for the treatment of psychiatric and neurological conditions, such as epilepsy 65. In chemical reprogramming protocols, HDAC inhibitors are applied to facilitate global transcriptional changes by overcoming the epigenetic barrier between different cell types 64. GSK3‐inhibitors like CHIR99021 are involved in the β‐catenin/Wnt signaling pathway and regulate cell division, proliferation, and stem cell maintenance. RepSox is a TGFβ signaling inhibitor that can replace the central reprogramming factor Sox2 by induction of Nanog expression. It additionally promotes mesenchymal‐to‐epithelial transition (MET) thereby facilitating the conversion of fibroblasts that are of mesenchymal origin 65, 66, 67. Other studies applied different combinations of pathway modulators, including A‐83‐01 (TGFβ inhibitor), purmorphamine (sonic hedgehog agonist), and thiazovivin (ROCK inhibitor) 60, 61, 68. However, a prerequisite for chemical reprogramming is that distinct cell populations need to be sensitive to the pharmacological active compounds that modulate cellular pathways involved in cell signaling, epigenetic status, metabolism, and transcriptional changes 64, 65. Another critical limitation of chemical reprogramming is the potential risk of chemical‐induced genotoxicity and cell toxicity as unwanted side effects, both of which need to be assessed for each compound in order to develop a reliable and robust system for direct conversion 69.

Other integration‐free systems used recombinant TAT‐SOX2 fusion protein to directly convert human fibroblasts toward the neural progenitor fate 33, or *in vitro* transcribed messenger RNA (IVT mRNA) encoding SOX2 to directly convert human cord blood‐derived mesenchymal stem cells into stably expandable iNSCs 34. Despite extensive troubleshooting, mRNA‐based reprogramming showed a rather low reprogramming efficiency due to time‐restricted influx of the exogenous mRNA.

In conclusion, lentiviral‐based systems are highly efficient in reprogramming, yielding reproducible results at relatively low cost, which makes them a reliable tool for basic and preclinical applications. However, it has to be taken into consideration that iPSCs or iNSCs reprogrammed *via* integrating methods potentially exhibit (epi‐)genetic aberrations. These can affect genomic integrity, including increased copy number variations (CNVs), accumulation of point mutations, dysregulation of imprinted genes, and aberrant methylation patterns 70. Consequently, generation of integration‐free iNSCs is highly desired to circumvent potential risks of mutagenesis in the context of cell therapy and clinical applications. Although a variety of protocols allow generating transgene‐free iNSCs by nonintegrating methods, reprogramming efficiencies differ significantly and each reprogramming method has specific limitations.

## Molecular and cellular characterization of iNSCs

*Ex vivo* and pluripotent stem cell‐derived neural stem cells (NSCs) share common properties, including self‐renewal potential, clonal growth, marker expression profile, epigenetic status, and multipotential differentiation capacity *in vitro* and *in vivo*. A key question to assess the outcome of direct conversion strategies is to which extent directly converted iNSCs recapitulate these characteristics 11, 71. As a matter of fact, the various protocols for the generation of iNSCs give rise to distinct iNSC populations that slightly differ in self renewal capacity, marker expression, and regional identity, as well as *in vitro* and *in vivo* differentiation potential. On the other hand, all iNSC populations have been reported to express pan‐neural markers, to be at least bipotential, and to show self‐renewal and clonal growth (Table 2). In order to demonstrate self‐renewal potential and clonal growth ability, iNSCs were either cultivated as primary and secondary neurospheres 33, 34, analyzed in colony formation assays 28, 33, 36, 39, 42, 48, 72, and/or passaged several times 11, 13, 14, 26, 30, 32, 33, 38, 57, 59, 60, 73. While Kim *et al*. 11 reported passage of their mouse iNSCs for 3–5 times only, more recent studies demonstrated maintenance of NSC characteristics for more than 30 passages 13, 14, 28, 34, 36, 38, 58, 73. Self‐renewal capacity was also assessed through immunocytochemical staining for proliferation markers, such as Ki‐67, in combination with key markers for NSCs 25, 26, 30, 34, 57, 59, 74. Among the various publications reporting iNSC generation, there is extensive accordance in terms of expression of *bona fide* neural stem cell markers, such as SOX1, SOX2, PAX6, NESTIN, CD133, and BLBP. However, iNSC populations also showed slight differences in their marker expression (Table 2). The various iNSC populations were generated and maintained in distinct media thereby also influencing their expression profiles. The neural expansion media were supplemented with either LIF and small molecules like CHIR99021, SB431542, purmorphamine, A83‐01, and/or ascorbic acid 25, 26, 48, or basic fibroblast growth factor (FGF2) and epidermal growth factor (EGF) 14, 30, 32, 34, 36, 38, 57, 58, 59, 60, 62, or even a combination of them 33, 35, 41. Two studies also included FGF4 in their neural expansion media 11, 42. Distinct culture medium supplements are not only well known to support NSC growth and self‐renewal, they also lead to a regional patterning along the anterior–posterior and dorso‐ventral axes during neurodevelopment 75. For example, CHIR99021, a potent Wnt agonist, leads to a posteriorization of NSCs in a concentration‐dependent manner, while purmorphamine, a sonic hedgehog agonist, has ventralizing effects on NSCs and their derivatives 75. Though a systematic side‐by‐side analysis is lacking, it is evident that the distinct iNSC populations show slightly different marker expression patterns and regional identities (Table 2). Several studies indicated a specific regional identity 14, 25, 28, 48, 59, while others suggested that the individual iNSC populations were of mixed regional identities 13, 73: Thier *et al*. 13 established mouse iNSCs with ventral fore‐, mid‐, and hindbrain identity, whereas generation of iNSC according to Tang *et al*. 73 led to fore‐, mid‐, and anterior hindbrain. Interestingly, Zhang *et al*. 60 reported that their iNSC population possesses an anterior identity at low passages, while at later passages iNSCs exhibited a clear posteriorization. This may reflect the *in vivo* situation, where caudalizing effects of the NSC pool are evident at more advanced developmental stages, when astrocyte and oligodendroglial precursors arise 75.

**Table 2:** Induced neural stem cells characterization. Pur, purmorphamine; CHIR, CHIR99021; SB, SB431542; Alk, Alk5 inhibitor II; AA, ascorbic acid; –, not reported or not applicable; ●, applicable

Reference	NSC cultivation	NSC marker						Regional identity	*In vitro* differentiation								*In vivo* differentiation			Electrophysiology
									Neuron					Synaptic marker	Astrocyte	Oligodendrocyte				
		Sox1	Sox2	Pax6	Nestin	CD133	BLBP		Glutamatergic	GABAergic	Serotonergic	Dopaminergic	Cholinergic				Neuron	Astrocyte	Oligodendrocyte	
Kim *et al*. 11	EGF, FGF2, FGF4	●		●		●		–		●					●		–	–	–	●
Han *et al*. 14	EGF, FGF2	●	●	●	●		●	Ventral posterior	●	●		●	●	●	●	●	●	●	●	●
Thier *et al*. 13	EGF, FGF2		●	●	●		●	Ventral fore/mid/hindbrain		●				●	●	●		●	●	●
Ring *et al*. 32	EGF, FGF2			●	●		●	–	●	●				●	●	●	●	●	●	●
Wang *et al*. 57	EGF, FGF2	●	●	●	●			–	●	●		●		●	●	●	●	●		●
Lu *et al*. 25	LIF, CHIR, SB	●	●	●	●		●	Dorsal hindbrain		●	●	●	●	●	●	●	●	●	●	●
Cheng *et al*. 59	EGF, FGF2		●	●	●		●	Ventral fore/midbrain	●	●				–	●	●	●	●	●	●
Lee *et al*. 30	EGF, FGF2		●	●	●	●		–	●	●		●		●	●	●	●	●		●
Mirakhori *et al*. 33	EGF, FGF2, SB, CHIR, Pur	●	●	●	●			Ventral mid/hindbrain		●		●			●	●	●	●		–
Tang *et al*. 73	LIF, CHIR, SB	●	●		●		●	Fore/mid/anterior hindbrain	●	●		●	●	●	●	●	–	–	–	●
Shahbazi *et al*. 36	EGF, FGF2	●	●		●			Rostral	●	●		●	●	●	●	●	●	●		●
Kim *et al*. 72	EGF, FGF2	●	●		●		●	–	–	–	–	–	–	–	●	●	●	●	●	●
Zhang *et al*. 60	EGF, FGF2		●	●	●		●	Anterior/posterior	●	●			●	●	●	●	●	●	●	●
Xiao *et al*. 38	EGF, FGF2,		●	●	●			–		●				●	●	●	●	●	●	●
Tang *et al*. 62	EGF, FGF2		●	●	●			–	–	–	–	–	–	–	●	●	–	–	–	●
Sheng *et al*. 48	CHIR, A83‐01, Pur, LIF, AA		●	●	●			Posterior ventral	●	●		●	●	●	●	●	●	●	●	●
Kim *et al*. 34	EGF, FGF2		●	●	●			–				●	●		●	●	–	–	–	–
Thier *et al*. 28	CHIR, Alk5, Pur, AA	●	●	●	●	●		Dorsal anterior hindbrain fate	●	●	●	●	●	●	●	●	●	●	●	●

## Differentiation potential of iNSCs

Similar to their *ex vivo* counterparts, iNSCs are multipotent stem cells giving rise to neurons and glial cells 71, 76, 77. However, the differentiation potential can vary depending on the iNSC population, as well as the *in vitro* differentiation protocol used (e.g., spontaneous, undirected, and directed *in vitro* differentiation approaches) (Table 2). Most iNSC populations were shown to be tripotential giving rise to neurons, astrocytes, and oligodendrocytes 13, 25, 28, 32, 34, 57, 58, 77. Yet, there seems to be a bias toward neurons and astroglia as differentiation into oligodendrocytes was less efficient 13, 14, 58. Other iNSC populations gave only rise to neurons and astrocytes 11, 26, 35, 78. However, this may reflect the current difficulties in generating oligodendroglial cells from stem cells. Regarding neuronal subtypes, iNSCs were shown to have the potential to differentiate into the main neuronal subtypes, including glutamatergic 28, 30, 32, 36, 48, 57, 59, 60, 73, 77, GABAergic 11, 13, 28, 30, 32, 36, 38, 48, 57, 59, 60, 77, dopaminergic 14, 25, 28, 30, 33, 34, 36, 48, 57, 73, serotonergic 25, 28, and cholinergic neurons 14, 25, 28, 34, 36, 48, 60, 73. However, direct comparison of these studies is difficult, since the authors used distinct iNSC populations and differentiation approaches. These facts obviously influence the outcome of the differentiation studies and might explain the apparent discrepancies in multipotency *in vitro*.

Besides analyses of the *in vitro* differentiation potential, the examination of iNSCs and their derivatives after transplantation is crucial to develop potential therapeutic applications. Several studies addressed both the differentiation and the tumor formation capacity of iNSCs *in vivo* by means of iNSC transplantation into the CNS of rodents. Remarkably, there were no tumors reported in any of the studies 25, 32, 60, 72, 79 suggesting safety of iNSC‐derived engraftments. Transplanted iNSCs were reported to survive, integrate into existing neuronal circuits, and differentiate into all three neural lineages 14, 25, 28, 32, 38, 48, 58, 59, 60. However, in some studies, transplanted iNSCs failed to differentiate into oligodendrocytes 30, 33, 57, 80. Lee *et al*. and Mirakhori *et al*. reported transplantation of human iNSCs into brains of newborn mice or rat pups, respectively 30, 33: While analyzing brains at different endpoints, both studies detected neurons and astrocytes, but no iNSC‐derived oligodendrocytes 30, 33. It might well be that in both studies, the brains were analyzed too early, given the fact that oligodendrocytic cells arise later than neurons and astrocytes during neurodevelopment 75. However, both studies showed that in vitro the same human iNSC populations could readily differentiate into oligodendroglial cells 30, 33. On the other hand, Han *et al*. transplanted mouse iNSCs into the subventricular zones of adult mice. After only 2 weeks, transplanted iNSCs were able to give rise to neurons, astrocytes, and oligodendroglial precursor cells 14. This might be due to the transplantation of iNSCs into subventricular zones, one of the stem cell niches of the adult brain, or the fact that mouse NSCs differentiate more rapidly than human NSCs 71, 76. More recently, human iNSC populations were engrafted into adult mouse brains, which were analyzed eight to eleven weeks later. In both studies, iNSCs differentiated into neurons, astrocytes, and oligodendrocytes 28, 48. Furthermore, engrafted iNSCs give rise to neurons that extend their projections into ipsi‐ and contralateral sites of the corpus callosum 48, and neurons derived from iNSCs can fire repetitive action potentials in preparations of acute brain slices 28. This corroborates that engrafted cells do not only survive and differentiate, but also integrate into existing neuronal circuits while exhibiting basic electrophysiological properties of functional neurons.

## Functional characterization of iNSC‐derived neurons

Most studies point toward functionality of iNSC‐derived neurons as judged by the presence of rapidly inactivating (TTX‐sensitive) sodium inward currents and (TEA‐sensitive) potassium outward currents (Table 2). However, maturity of these neurons varies considerably in respect of resting membrane potential and capability of firing repetitive action potentials 11, 13, 14, 25, 28, 30, 32, 33, 34, 36, 38, 48, 57, 58, 59, 60, 62, 73. Electrophysiological recordings performed at different time points of *in vitro* differentiation demonstrated the gradual functional maturation of the neurons upon the onset of differentiation 81. Moreover, several studies strongly suggest the presence of functional synapses, as indicated by their response to excitatory or inhibitory neurotransmitters, as well as of spontaneous postsynaptic currents, and the ability to (partially) abolish synaptic activity with specific blockers 11, 25, 28, 38, 42, 48, 57, 59, 75, 80. In addition to electrophysiological experiments, the ability to form synapses was also investigated through immunocytochemical approaches showing colocalization of pre‐ and postsynaptic markers like synapsin 1, vGLUT1, or PSD‐95 (Table 2 13, 14, 25, 28, 30, 32, 36, 38, 48, 57, 73, 75). However, the functionality of iNSC‐derived neurons shall be characterized in detail. For example, there is little information about pre‐ and postsynaptic markers coming from high‐ and super‐resolution microscopy. Moreover, many electrophysiological properties of iNSC‐derived neurons have not yet been investigated, such as the presence of functional voltage‐gated calcium channels, and the sodium and potassium channel subunit composition.

Nevertheless, the studies published thus far strongly suggest that iNSC‐derived neurons recapitulate most of the key features of their *in vivo* counterparts and that they are electrophysiological functional following to both *in vitro* and *in vivo* differentiation (Table 2). Of note, proper functionality is a prerequisite for using iNSCs and their derivatives in disease modeling, pharmacological compound, and toxicity screenings, as well as potential future cellular therapies.

## iNSCs as tools for disease modeling

NSCs hold great potential as a virtually unlimited source of patient‐specific neural cells for disease modeling and cell therapy 82, 83. The unique properties of iNSCs (self‐renewal, clonality, and multipotency) and the fact that they can be generated from somatic cellular sources make them powerful model systems to study human physiology and pathology *in vitro* and *in vivo*. iNSCs are particularly suited for characterizing neural cells in health and disease, as well as during neural development. For example, Hou *et al*. 37 have reported the generation of iNSCs derived from fibroblasts of patients suffering from Alzheimer's disease (AD), a fatal neurodegenerative disease of unknown etiology 84. Importantly, the iNSC‐derived neuronal cell cultures recapitulated cellular hallmarks of the disease, including increased levels of amyloid ß and phosphorylated TAU (pTAU) in neuronal cell bodies and processes 37. Moreover, treatment of patient‐derived neuronal cultures with GSK3b inhibitors resulted in a significant reduction of pTAU aggregates 37. The same group has also generated iNSCs from Huntington's disease (HD) patients. HD is a polyglutamine disease caused by CAG repeat expansion in the HTT gene 84. The authors showed that treatment of HD‐derived neuronal cells with an A2AR agonist reduced neuronal DNA damage in neurons, one of the hallmarks of HD 37. Machado–Joseph disease (MJD or spinocerebellar ataxia type III) is another type of polyglutamine diseases caused by unstable CAG repeats in the ATXN3 gene, resulting in an abnormal, aggregate‐prone form of ATAXIN‐3. ATAXIN‐3 aggregates cause degeneration of cells in the hindbrain 85. Sheng *et al*. 48 derived iNSCs from MJD patients, which successfully recapitulated the cellular and molecular hallmarks of the disease *in vitro*, such as the formation of SDS‐insoluble ataxin aggregates in neuronal cultures. More recently, Thier *et al*. established an iNSC‐based *in vitro* model for human congenital insensitivity to pain (CIP). CIP is a rare disorder in which patients do not experience any modalities of pain, except neuropathic pain 86. To recapitulate human CIP, they generated iNSCs deficient for the voltage‐gated sodium channel Nav1.9 (SCN9A^−/−^) using CRISPR/Cas9‐based genome editing 28. Differentiation of SCN9A^−/−^ iNSCs toward sensory neurons suggested a role for SCN9A in the differentiation process. Stimulation of sensory neurons with specific P2RX3 receptor agonists resulted in calcium influx, with SCN9A^−/−^ cultures exhibiting less activity and a paucity of synchronous calcium influx compared with the control neurons 28. This study illustrates the power of iNSC‐based disease modeling for PNS dysfunction and the first successful CRISPR/Cas9‐mediated genome editing in iNSCs. This strategy enables the straightforward introduction of desired mutations in iNSCs and circumvents laborious and costly iPSC derivation and subsequent differentiation into neural cells. iNSCs provide a valuable *in vitro* model of chemotherapy‐induced peripheral neuropathy (CIPN), a complication often seen in cancer patients, in which treatment causes axon dieback and nerve degeneration 87. Lee *et al*. 30 differentiated iNSCs into nociceptive sensory neurons that, upon treatment with chemotherapy drug taxol, exhibited a dose‐dependent reduction of their neurite length and a concomitant loss of viability. Taken together, these studies demonstrate that iNSCs are a highly valuable and versatile source for modeling human neural pathologies *in vitro*. As *in vitro* models, they can provide detailed mechanistic insight into these pathologies and, in combination with genome editing tools, can also help to prove genomic and functional relationships. Moreover, iNSC‐derived *in vitro* models could be used for pharmacological and toxicological assays, thereby complementing or reducing animal experiments. Furthermore, patient‐specific iNSCs would facilitate precision medicine, for example, enabling screens aiming at identifying alternative pharmacological treatments for ‘pharmacoresistant’ patients.

## Preclinical studies employing iNSCs for cell therapy

In comparison with iPSCs, iNSCs represent a more feasible and cost‐efficient source of NSCs for clinical applications 77. Several transplantation studies in animal models of human pathologies, including acute and chronic brain damage and brain cancer, have explored the therapeutic potential of iNSCs.

Already in 2012, Thier *et al*. pioneered the preclinical usage of mouse iNSCs in glial cellular replacement therapy by engrafting iNSCs into brains of neonatal myelin deficient rats. Two weeks after transplantation, the authors found iNSC‐derived astrocytes and oligodendrocytes in distinct brain areas with concomitant PLP formation 13. Hong *et al*. transplanted mouse iNSCs into a rat model of spinal cord injury, next to the injured site. The iNSC‐derived cells migrated and differentiated and, most importantly, restore damaged tissues reconstituting the local circuitry. Mouse iNSC‐derived neurons extended their axons and formed synapses with host neurons and, already 4 weeks after transplantation, there were beneficial effects like immunomodulation, neural protection, and angiogenesis of the spinal cord. Ten weeks after transplantation, rats showed partial reconstitution of their locomotor function 88. Two years later, the same group transplanted mouse iNSCs into a mouse model of acute stroke. The mice underwent middle artery occlusion (MCAO) surgery, a model for ischemic brain injury. MCAO mice engrafted with iNSCs showed improved survival rate and a significant functional recovery. Surprisingly, histological analysis performed 1 and 8 months after transplantation showed that the vast majority of the engrafted iNSCs differentiated into astroglial cells, while only few cells were expressing oligodendroglial markers. Further, they did not detect any iNSC‐derived neuronal cells suggesting that the beneficial effects of the iNSC engraftment could derive from a possible secretory activity 89. Another study published in 2015 reported transplantation of mouse iNSCs into the brains of MCAO rats 90. Engrafted cells migrated and differentiated, thereby reducing the lesion size and improving motor and sensory functions. In addition, the authors showed that transplanted mouse iNSCs secreted nerve growth factors and displayed enhanced activity of anti‐apoptotic pathways. Despite both studies showing that iNSC transplantation has beneficial effects, the former and the latter detected mainly iNSC‐derived astroglial cells 89 and neuronal cells in the MCAO rat brains 90, respectively, pointing toward different rescuing mechanisms. This discrepancy could stem from many factors, such as the methods employed to generate mouse iNSCs, transplantation into different species and brain regions, endpoints of analyses, and amount of transplanted cells. The therapeutic potential of iNSCs to ameliorate pathological phenotypes in mouse models of Parkinson's disease (PD) and Alzheimer's disease has been also explored. Choi *et al*. transplanted mouse iNSCs into the brains of toxin‐induced mouse models of PD. Cells engrafted next to the 6‐hydroxydopamine (6‐OHDA) injection site migrated into the striatum and the substantia nigra pars compacta, where they differentiated mainly into dopaminergic neurons. Restoration of brain tissue led to an enhanced functional recovery of the animals in behavioral assessment 77. Another study investigating the effects of iNSC engraftment in the 6‐OHDA‐induced mouse model of PD found restored dopamine production and improved motor behavior, despite low survival rates of engrafted cells 91. Yet, another study reported transplantation of mouse iNSCs into the hippocampus of the APP/PS1 mouse model of AD. One month after transplantation, behavioral assessment showed that transplanted mice performed significantly better in the Morris water maze test as compared to the control group. These results strongly suggest that transplanted mouse iNSCs can significantly improve spatial learning and memory of APP/PS1 AD mice 38. In addition, the authors transplanted mouse iNSCs into Aβ1–40 (amyloid β peptide 1–40)‐injured AD mouse model, which displays both learning and memory impairment similar to that of AD. Again, they transplanted mouse iNSCs into the hippocampus of Aβ1–40‐injured mice and run behavioral tests 2 months after transplantation. In line with their previous experiments, these results showed that transplanted mouse iNSCs significantly improved spatial learning and memory of Aβ1–40‐injured mice 38, though the underlying mechanisms remained unclear. More recently, Peruzzotti‐Jametti *et al*. were able to dissect the molecular mechanism whereby iNSCs deploy their immunomodulatory action. They transplanted human iNSCs into the brain of mice with experimental autoimmune myelin oligodendrocyte glycoprotein (MOG)‐induced encephalomyelitis (EAE), a mouse model of multiple sclerosis (MS). The authors demonstrated that human iNSC engraftment reduced pro‐inflammatory succinate in the cerebrospinal fluid, thereby decreasing mononuclear phagocyte infiltration and secondary CNS damage. The transplantation of iNSCs induced a significant and long‐lasting amelioration of EAE scores and led to a behavioral and pathological recovery. Notably, the succinate dependency of anti‐inflammatory response mounted by iNSCs was confirmed genetically using mouse cells devoid of succinate receptor 92.

Finally, Bago *et al*. used mouse iNSCs as a vehicle to deliver anticancer protein TRAIL in a mouse model of glioblastoma. They engrafted mouse xenograft models of human glioblastoma with iNSCs stably secreting TRAIL and confirmed homing of iNSCs to the tumors *in vivo*. Local TRAIL secretion induced apoptosis in human glioblastoma cells and, most importantly, iNSC‐derived engraftment significantly prolonged mouse survival. Thus, this approach might be useful for the generation of autologous cell‐based therapies for the treatment of patients with aggressive forms of brain cancer 93.

## Conclusions and perspectives

In the shadow of the mainstream iPSC technology, second‐generation reprogramming protocols have recently been developed to derive multipotent precursors/stem cells by direct conversion of somatic cells. In general, direct conversion allows a faster, more cost‐efficient shortcut to patient‐derived stem cells and yields also cells with a low tumorigenic potential. iNSCs exhibit clonal growth, virtually unlimited self‐renewal, and neural differentiation potential. Thus, iNSCs are functionally indistinguishable from NSCs derived from pluripotent stem cells and primary tissue. Various protocols for iNSC generation have been published, some involving either the Yamanaka or neurogenic TFs, others a mix of both. While robust derivation of iNSC from somatic cells is well established, more work is needed to take control of the regional identity of iNSCs.

Mechanistically, it is still debated whether direct conversion into iNSCs involves a transient pluripotent state. In fact, lineage tracing experiments have identified a pluripotent intermediate in some transdifferentiation protocols. Other protocols instead allow circumventing pluripotency during iNSC induction, indicating that the choice of TFs and delivery methods impact on the reprogramming trajectory. Single‐cell tracing will help to shed more light on the mechanisms of transdifferentiation 94. In any case, stably reprogrammed iNSCs are free of iPSC contaminants, making them suited for therapeutic applications. In this regard, nonintegrating methods make it possible to generate transgene‐free iNSCs. Unfortunately, the existing nonintegrating methods, such as small molecules, mRNA, or episomal transfection, yield reprogramming efficiencies lower than viral transduction. Yet, we foresee that the development of miniaturized microfluidic devices enabling massive scale‐down and parallelization of iNSC generation will further reduce the production costs 95 and promote a widespread clinical use.

Induced neural stem cells have proven extremely valuable to study human disease pathologies like Alzheimer's and Huntington's disease, and congenital insensitivity to pain. Moreover, they can be employed in pharmacological and toxicological screenings and this will help to reduce animal experimentation. Patient‐specific iNSCs will be instrumental in cost‐efficient precision medicine applications, such as screening for alternative pharmacological treatments for patients with intrinsic and acquired pharmacoresistance. Finally, preclinical studies indicate that iNSCs may revolutionize autologous cell therapy. For instance, iNSCs have been proven to ameliorate pathological symptoms in animal models of demyelinating diseases, spinal cord injury, acute stroke, Parkinson's disease, and multiple sclerosis. Also, allogenic iNSC banks matching the major transplant antigens present in most recipients, so that immunological rejection is minimized, might be considered. Given that PMBCs provide a robust basis for iNSC derivation 28, this could be accomplished by repurposing human leukocyte antigen‐matched blood banks.

In spite of preclinical studies giving encouraging results, there remain several important open questions. For example, do clinical effects vary when engrafting distinct iNSC populations? How is the site (cellular niche) of transplantation influencing the migration, differentiation, and grafting of iNSCs? What are the mechanisms mediating differentiation and grafting of transplanted cells? To what extent and how can researchers/clinicians control these mechanisms? How pure must a certain cell type be or is a predifferentiation of benefit? Future studies are needed to deepen our knowledge on iNSCs and help to exploit their potential as an unlimited and versatile autologous source of cells for modeling human neural pathologies and curative therapies.
